# Cardiovascular Disease Among Women Who Gave Birth to an Infant With a Major Congenital Anomaly

**DOI:** 10.1001/jamanetworkopen.2018.2320

**Published:** 2018-09-21

**Authors:** Eyal Cohen, Erzsébet Horváth-Puhó, Joel G. Ray, Lars Pedersen, Vera Ehrenstein, Nancy Adler, Simone Vigod, Arnold Milstein, Henrik Toft Sørensen

**Affiliations:** 1 Department of Pediatrics, The Hospital for Sick Children, University of Toronto, Toronto, Ontario, Canada; 2 Department of Clinical Epidemiology, Aarhus University Hospital, Aarhus, Denmark; 3 Institute of Health Policy, Management, and Evaluation, University of Toronto, Toronto, Ontario, Canada; 4 Department of Medicine, St Michael’s Hospital, University of Toronto, Toronto, Ontario, Canada; 5 Center for Health and Community, School of Medicine, University of California, San Francisco; 6 Reproductive Life Stages Program, Department of Psychiatry, Women’s College Hospital, Toronto, Ontario, Canada; 7 Clinical Excellence Research Center, Stanford University School of Medicine, Stanford, California; 8 Division of Epidemiology, Department of Health Research and Policy, Stanford University, Stanford, California

## Abstract

**Importance:**

Having a child with a major birth defect can be a life-changing and stressful event that may be associated with higher cardiovascular disease (CVD) risk, yet the long-term burden of CVD for the child’s mother is unknown.

**Objective:**

To assess whether mothers of an infant born with a major congenital anomaly are at higher risk of CVD compared with a comparison cohort.

**Design, Setting, and Participants:**

A population-based cohort study using individual-level linked registry data in Denmark included 42 943 women who gave birth to an infant with a major congenital anomaly between January 1, 1979, and December 31, 2013; and follow-up was conducted until 2015. A comparison group, comprising 428 401 randomly selected women, was 10:1 matched to each affected mother by maternal age, parity, and her infant’s year of birth. Data analyses were performed between November 1, 2017, and February 28, 2018.

**Exposures:**

Live birth of an infant with a major congenital anomaly.

**Main Outcomes and Measures:**

The primary outcome was a CVD composite outcome of acute myocardial infarction, coronary revascularization, or stroke. Secondary outcomes included individual components of the CVD composite and other cardiovascular outcomes, including unstable angina, congestive heart failure, atrial fibrillation, peripheral artery disease, ischemic heart disease, and aortic aneurysm. Cox proportional hazards regression analyses generated hazard ratios (HRs), adjusted for maternal demographic, socioeconomic, and chronic health indicators.

**Results:**

Median maternal age at baseline was 28.8 years (interquartile range, 25.3-32.5 years). After a median follow-up of 19.5 years (interquartile range, 9.9-27.6 years), 914 women whose infant had a major congenital anomaly experienced a CVD event (1.21 per 1000 person-years; 95% CI, 1.13-1.28 per 1000 person-years) vs 7516 women in the comparison group (0.99 per 1000 person-years; 95% CI, 0.97-1.01 per 1000 person-years), corresponding to an unadjusted HR of 1.23 (95% CI, 1.15-1.32), and an adjusted HR (aHR) of 1.15 (95% CI, 1.07-1.23). Women who gave birth to an infant with multiorgan anomalies had an even higher aHR (1.37; 95% CI, 1.08-1.72). Mothers of infants with a major anomaly also had an increased aHR of the individual components of the composite outcome and the other cardiovascular outcomes.

**Conclusions and Relevance:**

Women whose child had a major congenital anomaly experienced a 15% to 37% higher risk of premature cardiovascular disease. These women may benefit from targeted interventions aimed at improving their cardiovascular health.

## Key Points

**Question:**

Do mothers who give birth to an infant with a major congenital anomaly have increased cardiovascular risk?

**Findings:**

In this cohort study of 471 344 Danish women, mothers of infants born with a major congenital anomaly had a 15% increased risk of acute myocardial infarction, coronary revascularization, or stroke compared with women without an affected infant. This elevated risk rose to 37% among women who gave birth to more severely affected infants with multiorgan congenital anomalies.

**Meaning:**

Having a child with a major congenital anomaly was associated with a small but significantly increased cardiovascular risk in the mother.

## Teaser

This cohort study examines the risk for cardiovascular disease among women in Denmark who have given birth to children with major congenital anomalies.

## Introduction

Major congenital anomalies affect 2% to 5% of all births in the United States and Europe.^1,2,3^ Having a child with a severe chronic illness, such as a major anomaly, can be a life-changing event for the child’s mother, including high levels of chronic stress^4^ associated with providing care to a child with complex needs within the home setting.^5^ Such caregiving demands can also affect a woman’s ability to pursue a healthy lifestyle. Caregiving parents have been reported to have higher rates of chronic conditions, activity limitations, and poorer physical and mental health compared with parents of healthy children.^6,7,8,9^

Chronic stress is associated with cardiovascular disease,^10^ including acute myocardial infarction^11^; however, an association with stroke is less certain.^12^ Cohort studies conducted in the workplace suggest a 40% to 50% increased risk of coronary heart disease related to stress.^13,14^ Caregiver stress studies have been primarily conducted in older individuals with a limited duration of follow-up.^15^ Little is known about the health risks associated with maternal caregiving for children with a chronic condition. These risks should be examined, as women play a central role in child rearing, which may continue well beyond the early years of the child.

This matched cohort study was undertaken to evaluate whether the mother of an infant with a major congenital anomaly is at increased risk of cardiovascular disease (CVD). The association was further assessed by the nature of the congenital anomaly and by various CVD outcome subtypes.

## Methods

### Study Design, Setting, and Data Linkage

This was a cohort study with analyses completed between November 1, 2017, and February 28, 2018, using routinely collected data from national registries in Denmark, a country of 5.7 million people with universal health care access.^16^ We used the Medical Birth Registry to identify all women with a liveborn, singleton infant born between January 1, 1979, and December 31, 2013. The study was restricted to mothers who had a minimum of 2 years of health data available prior to the index birth and who remained alive and residing in Denmark for at least 1 year thereafter. The minimum 2-year lookback window permitted the exclusion of women with prior CVD, as well as to capture preexisting comorbid conditions, and the minimum of 1-year postpartum survival was set to exclude women with a CVD event as a complication of pregnancy. The Medical Birth Registry contains information on all births at week 22 or later occurring in Denmark. Linkage to a mother’s infant was possible through the Danish Civil Registration System, which assigns a unique number to all Danish residents, permitting unambiguous individual-level data linkage across all sources. Data in the Civil Registration System on migration and vital statistics are updated daily.^17,18^ The Medical Birth Registry was used to abstract registered information on numbers of parents and newborns, date of birth, singleton vs multiple births, gestational age, and various physical characteristics of the newborn.^19^

The study received the required approval from the Danish Data Protection Agency, which oversees the confidentiality of individual-level information in Danish registries and from the Hospital for Sick Children’s Research Ethics Board. Informed consent was not required for this registry-based study. The study followed the Strengthening the Reporting of Observational Studies in Epidemiology (STROBE) reporting guideline.

### Cohorts

#### Major Congenital Anomalies (Exposed) Cohort

Major congenital anomalies were identified through linkage to the Danish National Patient Registry.^20^ This registry has tracked all Danish hospitalizations since 1977 and all outpatient and emergency department visits since 1995. The Danish National Patient Registry contains admission and discharge data on all hospital contacts, using the *International Classification of Diseases, Eighth Revision* (*ICD-8*) until 1993, and *International Classification of Diseases, Tenth Revision* (*ICD-10*) since 1994. Congenital anomalies were defined using the European Surveillance of Congenital Anomalies (EUROCAT) classification system,^21^ with minor modification for use with Danish registry data.^16,22^ Among women with more than 1 live-birth pregnancy affected by a major anomaly, the first affected birth was designated as the index birth. All diagnostic and procedural codes used in the study are described in eTable 1 in the Supplement.

#### Comparison (Unexposed) Cohort

For each mother in the major congenital anomaly cohort, we randomly sampled up to 10 women in the Civil Registration System who had a singleton live birth without a congenital anomaly. The women were matched by maternal age, year of the infant’s birth, and parity (1, 2, and ≥3 children).

### Study Outcomes

The primary CVD outcome, starting at 365 days after the index delivery date, was a composite of a hospitalization for myocardial infarction, coronary artery revascularization by bypass graft or percutaneous intervention, or acute stroke (eTable 1 in the Supplement). Secondary outcomes, also starting at 365 days after the index delivery date, included each component of the primary CVD outcome, as well as hospitalization for unstable angina, congestive heart failure, atrial fibrillation, peripheral artery disease, ischemic heart disease, or aortic aneurysms.

### Covariates

Other study variables included maternal age at delivery, marital status, and immigration status, which were obtained from the Civil Registration System at the time of the index birth. Income quartile (available starting in 1980) and level of education (available starting in 1981) were obtained at the time of the index birth from Statistics Denmark.^23,24^ Earlier cohort participants (1979-1980) were assigned to income and educational level categories based on the earliest available data. The Medical Birth Registry provided information on previous live births, stillbirths, congenital anomalies, and any complication arising during the index pregnancy, including both placental syndromes (preeclampsia, gestational hypertension, or placental abruption/infarction) and nonplacental syndromes (intrauterine hypoxia/birth asphyxia, uterine rupture, umbilical cord prolapse, vasa previa, amniotic fluid embolism, and fetal hemorrhage) (eTable 1 in the Supplement). Maternal medical history was ascertained from the Danish National Patient Registry and summarized using a modified Charlson Comorbidity Index score that excluded diabetes, chronic hypertension, and alcohol-related liver disease.^25^ Diabetes and alcohol use are important correlates of both congenital anomalies and poor maternal health^26,27,28,29,30^ and thus were each handled as a separate covariate, as was chronic hypertension. Maternal smoking (data available from 1991 and thereafter) and body mass index (data available from 2004 and thereafter) were used only in additional analyses.

### Statistical Analysis

Maternal cohorts were followed up for outcomes from 365 days after the date of delivery until an outcome, death, emigration, or study end (December 31, 2014), whichever occurred first. In the main analysis, cumulative incidence curves were plotted for women who gave birth to an infant with a major congenital anomaly and their matched counterparts in the comparison cohort (the referent). Cox proportional hazards regression analyses accounting for the matched design compared the risk of each outcome between the 2 cohorts, expressed as hazard ratios (HRs) and 95% CIs. Hazard ratios were adjusted for covariates associated with baseline maternal demographics (marital status, immigration status), socioeconomic status (income quartile, educational level), health conditions prior to the index birth (diabetes, chronic hypertension, modified Charlson Comorbidity Index score, history of alcohol-related disease), previous spontaneous abortion, and pregnancy complications. The proportional hazards assumption was assessed graphically using –ln(-ln[survival]) vs ln(analysis time) and no major violations were observed. The main model was also reevaluated by CVD subtypes (additional analysis 1). All models were censored on death; however, fatal cardiovascular events, with a CVD event recorded in the Danish National Patient Registry on the date of death or beforehand, were included in all analyses.

Childhood multiorgan complex chronic disease has been associated with more severe consequences than diseases affecting a single organ.^31,32^ Thus, to assess a potential dose-response association between the exposure and the outcome, major congenital anomalies were subdivided into those affecting more than 1 organ system (eg, both congenital heart disease and congenital renal anomaly) and those affecting a single organ system (eg, isolated congenital heart disease), and risks were compared with the matched comparison cohort (additional analysis 2).

The main model was stratified by (1) duration of follow-up (0 to <10 years, 10 to <20 years, and 20-36 years); (2) year of delivery (1979-1993 [*ICD-8*], 1994-2004 [*ICD-10* era with complete information about smoking], and 2004-2013 [*ICD-10* era with complete information about both smoking and body mass index]), (3) infant prematurity (<37 and ≥37 weeks), and (4) infant death in the first year of life (additional analysis 3).

Furthermore, the cohorts were restricted to women without a personal history of a congenital cardiac anomaly^33^ and the main model was rerun (additional analysis 4). Because congenital and acquired heart disease may share some common genetic pathways,^34^ women without a personal history of congenital cardiac anomaly were reevaluated, further subdivided by those whose infant had a solitary cardiac vs noncardiac birth anomaly (additional analysis 5). Finally, to assess for potential selection bias introduced by the matching process, we ran the primary analyses with matching dissolved, stratified by year of delivery (additional analysis 6). All analyses were conducted using SAS, version 9.4 (SAS Institute Inc).

## Results

**Figure 1.**
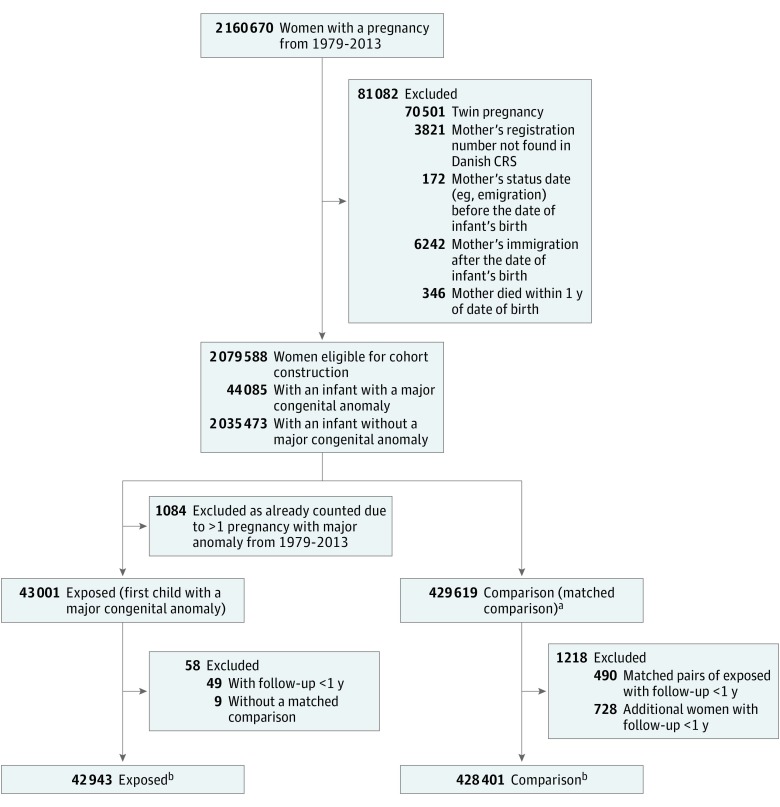
Cohort Derivation CRS indicates Civil Registration Service. ^a^A total of 430 000 women were expected; therefore, 391 (0.09%) without complete 1:10 match (eg, extremes of maternal age or party). ^b^For the primary outcome (acute cardiovascular events), an additional 50 women in the exposed group and 350 in the comparison cohort with a history of an acute cardiovascular were excluded for a total of 42 893 in the exposed and 428 051 in the comparison cohorts.

Of 2 079 588 eligible women during the study period, 44 085 (2.1%) gave birth to an infant with a major congenital anomaly (Figure 1). After further exclusions, 42 943 mothers remained in the exposed cohort, of whom 3842 (8.9%) had a birth affected by a multiorgan congenital anomaly and 39 101 (91.1%) had a birth affected by a single-organ congenital anomaly. Matched to these women were 428 401 mothers who formed the comparison (unexposed) cohort. The median (interquartile range [IQR]) age at baseline of the women in the study was 28.8 (25.3-32.5) years (Table 1). A majority of the women were married (59.1%), and 48.8% were primiparous.

**Table 1.** Characteristics of Women Included in Cohort and Their Infants

Characteristic	Cohort, No. (%)^a^	
	Major Congenital Anomaly (n = 42 943)	Comparison (n = 428 401)
**Mother**		
Age at delivery, median (IQR), y	28.8 (25.3-32.5)	28.8 (25.3-32.5)
Parity		
1	20 940 (48.8)	208 950 (48.8)
2	14 441 (33.6)	144 095 (33.6)
≥3	7562 (17.6)	75 356 (17.6)
Year of delivery		
1979-1993	20 246 (47.1)	202 209 (47.2)
1994-2003	11 197 (26.1)	111 695 (26.1)
2004-2013	11 500 (26.8)	114 497 (26.7)
Married/registered partnership^b^	25 072 (58.4)	253 752 (59.2)
Immigrated	2943 (6.9)	28 853 (6.7)
Lowest income quartile^c^	10 723 (25.0)	103 182 (24.1)
Postsecondary education^d^	10 427 (24.3)	111 905 (26.1)
Pregnancy history		
Spontaneous abortions	6498 (15.1)	58 578 (13.7)
Stillbirths	405 (0.9)	2802 (0.7)
Index pregnancy complications		
Placental^e^	2267 (5.3)	18 356 (4.3)
Nonplacental^f^	114 (0.3)	859 (0.2)
Medical history		
Diabetes	1463 (3.4)	9591 (2.2)
Chronic hypertension	204 (0.5)	1329 (0.3)
Alcohol-related diseases	521 (1.2)	3732 (0.9)
Hypercholesterolemia	30 (0.1)	170 (<0.1)
Modified Charlson Comorbidity Index score^g^		
1	719 (1.7)	6189 (1.4)
≥2	259 (0.6)	2074 (0.5)
Maternal smoking^h^	5863/26 829 (21.9)	54 177/267 447 (20.3)
Maternal BMI^i^		
<25	6876 (59.8)	72 507 (63.3)
25-29	2056 (17.9)	19 737 (17.2)
≥30	1953 (17.0)	16 146 (14.1)
Duration of follow-up, median (IQR), y^j^	19.4 (9.9-27.6)	19.5 (9.9-27.6)
**Infant**		
Sex		
Male	25 475 (59.3)	218 384 (51.0)
Female	16 404 (38.2)	209 382 (48.9)
Unknown	1064 (2.5)	635 (0.1)
Birth weight, g		
≤2500	5916 (13.8)	17 968 (4.2)
>2500-4000	31 368 (73.0)	344 408 (80.4)
>4000	5551 (12.9)	64 899 (15.1)
Unknown	108 (0.3)	1126 (0.3)
Gestational age		
<37 wk	5455 (12.7)	18 811 (4.4)
Unknown	1245 (2.9)	11 814 (2.8)
Apgar score <7 at 5 min	1793 (4.2)	6497 (1.5)
Died before end of observation period	936 (2.2)	1699 (0.4)

Abbreviations: BMI, body mass index (calculated as weight in kilograms divided by height in meters squared); IQR, interquartile range.

^a^ All data reflect measures at time of child’s birth unless otherwise indicated.

^b^ Missing data on 6192 (14.4%) in the exposed group and 59 911 (14.0%) in the comparison cohort.

^c^ Data available starting in 1980; for mothers who gave birth in 1979, data from 1980 were used for the baseline. Missing data on 265 (0.6%) in the exposed group and 2519 (0.6%) in the comparison cohort.

^d^ Data available starting in 1981; for mothers who gave birth in 1979-1980, data from 1981 were used for the baseline. Missing data on 2158 (5.0%) in the exposed group and 20 352 (4.8%) in the comparison cohort.

^e^ Includes preeclampsia, gestational or unspecified hypertension, placental abruption, or placental infarction.

^f^ Includes intrauterine hypoxia and birth asphyxia, uterine rupture, umbilical cord prolapse, vasa previa, amniotic fluid embolism, fetal-maternal hemorrhage, or chorioamnionitis.

^g^ Includes comorbid conditions with various weights. The modified index excludes diabetes and liver diseases associated with alcohol use.

^h^ Data only available starting in 1991 (26 829 women in the exposed group and 267 447 women in the comparison cohort). Missing data on 1484 (5.5%) in the exposed group and 11 421 (4.3%) in the comparison cohort.

^i^ Data only available starting in 2004 (11 500 women in the exposed group and 114 497 women in the comparison cohort). Missing data on 615 (5.3%) in the exposed group and 6107 (5.3%) in the comparison cohort.

^j^ Follow-up time only measured at 1 year after the birth of the child.

No appreciable differences were seen between the affected and comparison cohorts of women in demographics (year of birth, age at delivery, marital status, immigration status, educational level, and income), pregnancy history (parity, history of spontaneous abortions and stillbirths), index pregnancy complications, and maternal medical history. Among infants, the prevalence of low birth weight was more common in the major congenital anomalies group (5916 [13.8%]) than in the comparison group (17 968 [4.2%]), as was prematurity (5455 [12.7%] vs 18 811 [4.4%]), low 5-minute Apgar score (1793 [4.2%] vs 6497 [1.5%]), and mortality during the observation period (936 [2.2%] vs 1699 [0.4%]) (Table 1).

**Figure 2.**
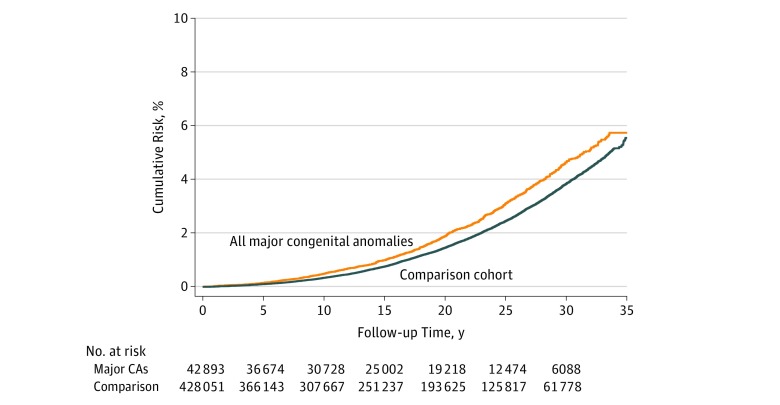
Development of the Cardiovascular Disease Composite Outcome of a Hospitalization Mothers whose infants had a major congenital anomaly (CA) and those in the comparison cohort (main analysis*)* hospitalized for acute myocardial infarction, coronary artery revascularization, or acute stroke.

After a median (IQR) follow-up of 19.4 (9.9-27.6) years, 914 mothers of infants with major congenital anomalies experienced a primary composite CVD event (1.21 per 1000 person-years; 95% CI, 1.13-1.28 per 1000 person-years) vs 7516 women in the comparison group (0.99 per 1000 person-years; 95% CI, 0.97-1.01 per 1000 person-years) (main analysis) (Figure 2 and Table 2). This finding corresponded to an unadjusted HR of 1.23 (95% CI, 1.15-1.32) and an adjusted HR (aHR) of 1.15 (95% CI, 1.07-1.23).

**Table 2.** Development of the Primary Cardiovascular Disease Composite Outcome of Hospitalization for the Main Analysis and Additional Analysis 1^a^

Outcome	No. of Events		Incidence Rate per 1000 Person-Years (95% CI)			HR (95% CI)	
	Exposed^b^	Comparison^c^	Exposed	Comparison	Rate Difference	Unadjusted	Adjusted^d^
Primary outcome^e^	914	7516	1.21 (1.13 to 1.28)	0.99 (0.97 to 1.01)	0.22 (0.14 to 0.30)	1.23 (1.15 to 1.32)	1.15 (1.07 to 1.23)
Primary outcome components							
Myocardial infarction	266	2224	0.35 (0.31 to 0.39)	0.29 (0.28 to 0.30)	0.06 (0.01 to 0.10)	1.21 (1.06 to 1.37)	1.12 (0.98 to 1.28)
CABG or PCI	235	1911	0.31 (0.27 to 0.35)	0.25 (0.24 to 0.26)	0.06 (0.02 to 0.10)	1.24 (1.08 to 1.42)	1.12 (0.97 to 1.29)
Stroke	592	4841	0.78 (0.72 to 0.84)	0.64 (0.62 to 0.65)	0.14 (0.08 to 0.21)	1.23 (1.13 to 1.34)	1.16 (1.06 to 1.27)
Other cardiovascular outcomes							
Unstable angina	787	6989	1.04 (0.96 to 1.11)	0.92 (0.90 to 0.94)	0.12 (0.04 to 0.19)	1.13 (1.05 to 1.22)	1.08 (1.00 to 1.16)
Congestive heart failure	219	1574	0.29 (0.25 to 0.32)	0.21 (0.20 to 0.22)	0.08 (0.04 to 0.12)	1.41 (1.22 to 1.62)	1.28 (1.10 to 1.48)
Atrial fibrillation	374	3277	0.49 (0.44 to 0.54)	0.43 (0.41 to 0.44)	0.06 (0.01 to 0.11)	1.15 (1.03 to 1.28)	1.10 (0.99 to 1.23)
Peripheral artery disease	255	1887	0.33 (0.29 to 0.38)	0.25 (0.24 to 0.26)	0.09 (0.04 to 0.13)	1.36 (1.19 to 1.55)	1.25 (1.09 to 1.43)
Ischemic heart disease	788	7017	1.04 (0.96 to 1.11)	0.92 (0.90 to 0.94)	0.12 (0.04 to 0.19)	1.13 (1.05 to 1.22)	1.07 (1.00 to 1.16)
Aortic aneurysm	14	109	0.02 (0.01 to 0.03)	0.01 (0.01 to 0.02)	0.00 (−0.01 to 0.01)	1.28 (0.73 to 2.23)	1.28 (0.73 to 2.25)

Abbreviations: CABG, coronary artery bypass graft; HR, hazard ratio; PCI, percutaneous coronary intervention.

^a^ Main analysis: acute myocardial infarction, coronary artery revascularization, or acute stroke; additional analysis: other various cardiovascular outcomes.

^b^ Number at risk excludes mothers with a history of an event prior to the infant’s birth: primary composite outcome (42 893), myocardial infarction (42 939), stroke (42 898), CABG or PCI (42 942), unstable angina (42 923), congestive heart failure (42 924), atrial fibrillation (42 922), peripheral artery disease (42 931), ischemic heart disease (42 920), and aortic aneurysm (42 940).

^c^ Number at risk excludes mothers with history of event prior to infant’s birth: primary composite outcome (428 051), myocardial infarction (428 366), stroke (428 092), CABG or PCI (428 386), unstable angina (428 279), congestive heart failure (428 316), atrial fibrillation (428 237), peripheral artery disease (428 280), ischemic heart disease (428 266), and aortic aneurysm (428 391).

^d^ Adjusted for matching variables and maternal demographics (marital status, immigration status), socioeconomic status (income quartile, educational level), previous maternal health status (diabetes, modified Charlson Comorbidity Index score, chronic hypertension, history of alcohol-related disease, and depression), previous spontaneous abortion, and pregnancy complications.

^e^ Composite cardiovascular disease outcome of hospitalization for myocardial infarction, CABG or PCI, or acute stroke.

Individual CVD outcomes were also more common in women with a child born with a major anomaly than in women in the comparison group (additional analysis 1) (Table 2). Of the composite outcome components, HRs were all increased, but the aHR was significantly increased only for stroke (1.16; 95% CI, 1.06-1.27). For the other noncomposite CVD outcomes, the aHR was consistently elevated, with a wide 95% CI for aortic aneurysm (Table 2).

Relative to the comparison cohort, women who gave birth to a child with a multiorgan congenital anomaly had a more pronounced risk of CVD (aHR, 1.37; 95% CI, 1.08-1.72) than the risk among those with an offspring with a single-organ congenital anomaly (aHR, 1.13; 95% CI, 1.05-1.22) (additional analysis 2) (eFigure in the Supplement; Table 3). Stratification of the main model by duration of follow-up, era of the index birth, preterm birth, and infant death within 1 year showed a persistence of the main effect, with widended 95% CIs among strata with relatively few outcome events (prematurity, infant death, and more contemporary birth years) (additional analysis 3) (Table 3).

**Table 3.** Development of the Cardiovascular Disease Composite Outcome of Hospitalization for Additional Analysis 2 and 3^a^

Analysis	No. of Events/No. of Women at Risk		Incidence Rate per 1000 Person-Years (95% CI)			HR (95% CI)	
	Exposed	Comparison	Exposed	Comparison	Rate Difference	Unadjusted	Adjusted^b^
Overall	914/42 893	7516/428 051	1.21 (1.13 to 1.28)	0.99 (0.97 to 1.01)	0.22 (0.14 to 0.30)	1.23 (1.15 to 1.32)	1.15 (1.07 to 1.23)
Additional analysis 2							
Major congenital anomaly subgroup							
Multiorgan	90/3837	609/38 303	1.42 (1.13 to 1.71)	0.95 (0.87 to 1.03)	0.47 (0.17 to 0.77)	1.50 (1.20 to 1.87)	1.37 (1.08 to 1.72)
Single-organ	824/39 056	6907/389 748	1.19 (1.10 to 1.27)	0.99 (0.97 to 1.02)	0.19 (0.11 to 0.28)	1.20 (1.12 to 1.30)	1.13 (1.05 to 1.22)
Additional analysis 3							
Follow-up time, y^c^							
0 to 10	174/42 893	1189/428 051	0.47 (0.40 to 0.54)	0.32 (0.31 to 0.34)	0.15 (0.08 to 0.22)	1.47 (1.25 to 1.72)	1.30 (1.10 to 1.54)
>10 to 20	343/30 728	2766/307 667	1.37 (1.23 to 1.52)	1.10 (1.06 to 1.14)	0.27 (0.12 to 0.42)	1.25 (1.12 to 1.40)	1.16 (1.03 to 1.30)
>20 to 35	397/19 218	3561/193 625	2.81 (2.54 to 3.09)	2.50 (2.42 to 2.58)	0.32 (0.03 to 0.60)	1.13 (1.02 to 1.26)	1.08 (0.98 to 1.21)
Year of birth							
1979 to 1993	736/20 232	6218/202 133	1.37 (1.27 to 1.47)	1.15 (1.12 to 1.18)	0.22 (0.11 to 0.32)	1.19 (1.11 to 1.29)	1.13 (1.05 to 1.22)
1994 to 2003^d^	142/11 178	1083/111 605	0.86 (0.72 to 1.01)	0.66 (0.62 to 0.70)	0.21 (0.06 to 0.35)	1.33 (1.11 to 1.58)	1.19 (0.99 to 1.43)
2004 to 2013^e^	36/11 483	215/114 313	0.64 (0.43 to 0.85)	0.38 (0.33 to 0.43)	0.26 (0.04 to 0.47)	1.72 (1.20 to 2.45)	1.36 (0.92 to 2.02)
Gestational age at birth, wk^f^							
<37	160/5433	488/18 772	1.86 (1.57 to 2.15)	1.54 (1.40 to 1.67)	0.32 (0.00 to 0.64)	1.23 (1.03 to 1.47)	1.15 (0.96 to 1.37)
≥37	688/36 215	6573/397 469	1.08 (1.00 to 1.16)	0.95 (0.92 to 0.97)	0.13 (0.05 to 0.22)	1.14 (1.05 to 1.23)	1.08 (1.00 to 1.17)
Infant death by 1 y	13/360	≤5^g^/150	2.12 (0.97 to 3.27)	1.02 (−0.13 to 2.17)	1.10 (−0.53 to 2.73)	2.00 (0.56 to 7.11)	2.16 (0.58 to 7.99)

Abbreviation: HR, hazard ratio.

^a^ Additional analysis 2: acute myocardial infarction, coronary artery revascularization, or acute stroke, evaluating mothers whose infant had a multiorgan major congenital anomaly; additional analysis 3: stratified by duration of follow-up, era of the index birth, preterm birth, and infant death within 1 year.

^b^ Adjusted for maternal demographics (marital status, immigration status), socioeconomic status (income quartile, educational level), previous maternal health (diabetes, modified Charlson Comorbidity Index score, chronic hypertension, history of alcohol-related disease, and depression), previous spontaneous abortion, and pregnancy complications.

^c^ Follow-up time begins at 1 year after the birth of the child.

^d^ Additional covariate adjusted for in 1994-2003 includes maternal smoking history. Without this additional covariate, the adjusted HR is 1.18 (95% CI, 0.98-1.42).

^e^ Additional covariates adjusted for in 2004-2013 include maternal smoking history and body mass index. Without these additional covariates, the adjusted HR is 1.38 (95% CI, 0.94-2.04).

^f^ Excludes mothers of infants with unknown gestational age.

^g^ Precise number supressed owing to small cell size.

After exclusion of women who had a personal history of congenital heart disease, the aHR for the CVD composite outcome was 1.15 (95% CI, 1.07-1.23) (additional analysis 4). Of these remaining women, the aHR was similar if they had an infant with a single major cardiac anomaly (1.15; 95% CI, 0.97-1.37) or a single major noncardiac anomaly (1.13; 95% CI, 1.04-1.23) (additional analysis 5). There was no appreciable difference in the aHRs when matching was dissolved (additional analysis 6) (eTable 2 in the Supplement).

## Discussion

A 15% relative increase in the risk of CVD events was observed among women whose live-born infant was affected by a major congenital anomaly. The risk was more pronounced, rising to a 37% increased risk following the birth of a child with a multiorgan congenital anomaly. Given the relatively young age of the cohort (median age at end of follow-up <50 years) and the presence of maternal risk even within the first 10 years of follow-up, these findings suggest a risk of premature cardiovascular disease in mothers of infants born with a major congenital anomaly.

The study findings are in keeping with a previous Danish study that demonstrated a 26% higher risk of death from cardiovascular disease among women whose child was affected by a major congenital anomaly.^16^ In a study of bereaved mothers and fathers, the death of a child was associated with a comparable increased risk of myocardial infarction in the exposed parents.^35^ This study extends these findings to CVD diagnoses, which can substantially affect current and future health and well-being. The use of validated diagnostic codes in this study to ascertain outcomes helps to validate these previous reports, because cause of death can be misclassified or incomplete in the absence of an autopsy.^36^

Stress is 1 plausible mediating factor between having a child with major congenital anomalies and poor cardiovascular health in the mother. Mothers caring for chronically ill children report experiencing chronic stress^37,38,39^ and rate their own health poorly compared with mothers of generally healthy children.^6,7,8,9^ Affected mothers also tend to exhibit impaired ability to suppress proinflammatory signals, such as interleukin-6,^40^ and biomarkers of advanced cell aging, including low telomerase activity and shorter telomere length.^41^ Animal and human studies of allostatic load have linked chronic stress exposure to health-damaging behaviors and adverse physiologic changes that increase cardiovascular risk.^42^ Other epidemiologic studies have found that cardiovascular risk is associated with chronic stressful situations.^43,44,45^ Furthermore, in the present study, higher risk was observed in women who gave birth to infants with more severe anomalies (multiorgan congenital anomalies) who likely pose greater caregiving challenges.

The association between having a child with a major congenital anomaly and subsequent CVD risk in the mother can also be partially explained by an increased risk of classic cardiovascular risk factors, such as diabetes and hypertension, in women who delivered a child with a major congenital anomaly. Although the absolute differences between the groups for these risk factors at baseline were not large, adjustment for these and other potential risk factors attenuated the HRs observed herein. Other unmeasured factors, including genetic (eg, family history of premature CVD) or behavioral (eg, poor diet, sedentary behavior, smoking, and/or obesity) variables, may affect the risk of both major congenital anomalies^46^ and CVD risk.^47,48^ Although we attempted to account for as many maternal factors as possible and conducted additional analyses to account for others (eg, excluding a personal history or infant exposure of congenital heart disease), some (eg, smoking and body mass index) were available for only part of the study period and thus left truncated, some were likely underestimated or too rare to include in the final model (eg, hypercholesterolemia), and others, such as dietary history,^49,50,51^ hereditary factors, and caregiver support, were not available.

Previous studies of cardiovascular health among women caregivers have focused primarily on caring for dependent adults (mainly elderly individuals) and have shown mixed findings. Some studies suggest an increased risk of coronary heart disease in caregivers of sick spouses, but not sick parents.^52^ Other studies report no significant association between caregiving and coronary artery disease.^15^ This discrepancy may occur because many family caregivers report little or no strain from caregiving or may be the result of selection bias, as healthy people are more likely to assume caregiving roles and/or may obtain health benefits from caregiving roles.^53^

In contrast, mothers who care for a child with a chronic condition appear to fare less well. One explanation may be the degree of time commitment required to rear such a child. Even among women who care for a healthy child, an association between hours of overall caregiving and coronary heart disease has been described.^54^ Other explanations for why findings for caregivers of children may differ from those for caregivers of adults include the younger age of the children’s caregivers, the type of care needed, the extent of choice in taking on a caregiving role, and/or the lack of preparation for the role. In addition, given relatively low mortality rates for children with complex chronic conditions compared with those occurring in adults,^55^ the duration of caregiving required for children is likely much longer.

### Strengths and Limitations

Our study’s population-based cohort design may have helped to minimize selection bias, using comprehensive linked registry data with robust follow-up information. As with any study using diagnostic coding data, misclassification of exposure, outcomes, and/or covariates is a potential risk. However, the exposure—birth of a child with a major congenital anomaly—occurred at a discrete time and was identified by a well-defined set of diagnostic codes.^2^ Substantial misclassification of the study outcomes is unlikely in Danish registries.^56,57,58^ Although CVD is relatively uncommon in women of childbearing age, the present study had sufficient population size, outcome events, and years of follow-up to provide stable estimates of the risks of the primary CVD outcome. Estimates of risks for less-common outcomes (eg, aortic aneurysm) were not as stable.

Potential confounding was reduced by matching exposed mothers with their comparison group on parity, maternal age, and year of delivery, and by adjusting for many covariates in the risk models. However, some key risk factors were not available, such as cholesterol levels, exercise, and family history of cardiovascular disease; in addition, others were left truncated and only used in subanalyses, such as smoking and body mass index. All covariates herein were handled as fixed-in-time variables. Thus, we could not consider any dynamic changes in a variable that might heighten or reduce maternal stress; provide a more-specific mechanistic explanation for the observations herein (eg, that stress leads to cardiovascular risk via its association with classic cardiovascular risk factors, such as such as smoking or hypertension, and may provide targets for prevention); or that describe the health trajectory of a child born with a major congenital anomaly. There was also no meaningful way to quantify caregiver requirements. Individuals in Denmark benefit from universal free health care, with relatively generous family assistance.^59^ In other countries, such as those with fewer health and social service supports, the findings may differ and be more pronounced.

## Conclusions

Women whose child had a major congenital anomaly experienced a 15% to 37% higher associated risk of premature CVD in this study. These women may benefit from targeted interventions aimed at improving their cardiovascular health.

## Notes

Supplement. **eTable 1.** Diagnostic and Procedural Codes Used in the Study, With Relevant References for Validation Studies **eTable 2.** Development of the Cardiovascular Disease Composite Outcome of Hospitalization for Acute Myocardial Infarction, Coronary Artery Revascularization, or Acute Stroke, Evaluating Mothers Whose Infant Had a Major Congenital Anomaly With Matching Dissolved Stratified by Duration of Follow-up (Additional Analysis 6) **eFigure.** Development of the Cardiovascular Disease Composite Outcome of a Hospitalization for Acute Myocardial Infarction, Coronary Artery Revascularization, or Acute Stroke, Evaluating Mothers Whose Infants Had a Multi-Organ or Single-Organ Major Congenital Anomaly and Those in the Comparison Cohort (Additional Analysis 2) **eReferences**
