# Error in Byline

**DOI:** 10.1001/jamanetworkopen.2019.2266

**Published:** 2019-03-29

In the Original Investigation titled “Cardiovascular Disease Among Women Who Gave Birth to an Infant With a Major Congenital Anomaly,”^1^ published September 21, 2018, there was an error in the author byline. The Author Affiliations and Author Contributions sections incorrectly listed the last name of Henrik Toft Sørensen, MD, DMSc, PhD, as “Toft Sørensen.” The proper listing is “Sørensen.” This article has been corrected.^1^
