# Supplementary material for: Cardiovascular Disease Among Women Who Gave Birth to an Infant With a Major Congenital Anomaly
Source: JAMA Netw Open. 2018 Sep 21;1(5):e182320. doi: 10.1001/jamanetworkopen.2018.2320 (PMC6324496; doi:10.1001/jamanetworkopen.2018.2320)
Supplement: Supplement. — eTable 1. Diagnostic and Procedural Codes Used in the Study, With Relevant References for Validation Studies eTable 2. Development of the Cardiovascular Disease Composite Outcome of Hospitalization for Acute Myocardial Infarction, Coronary Artery Revascularization, or Acute Stroke, Evaluating Mothers Whose Infant Had a Major Congenital Anomaly With Matching Dissolved Stratified by Duration of Follow-up (Additional Analysis 6) eFigure. Development of the Cardiovascular Disease Composite Outcome of a Hospitalization for Acute Myocardial Infarction, Coronary Artery Revascularization, or Acute Stroke, Evaluating Mothers Whose Infants Had a Multi-Organ or Single-Organ Major Congenital Anomaly and Those in the Comparison Cohort (Additional Analysis 2) eReferences [file jamanetwopen-1-e182320-s001.pdf]

Supplementary Online Content

Cohen E, Horváth-Puhó E, Ray JG, et al. Cardiovascular disease among women who gave birth to an infant with a major congenital anomaly. *JAMA Netw Open*. 2018;1(5):e182320. doi:10.1001/jamanetworkopen.2018.2320

eTable 1. Diagnostic and Procedural Codes Used in the Study, With Relevant References for Validation Studies

eTable 2. Development of the Cardiovascular Disease Composite Outcome of Hospitalization for Acute Myocardial Infarction, Coronary Artery Revascularization, or Acute Stroke, Evaluating Mothers Whose Infant Had a Major Congenital Anomaly With Matching Dissolved Stratified by Duration of Follow-up (Additional Analysis 6)

eFigure. Development of the Cardiovascular Disease Composite Outcome of a Hospitalization for Acute Myocardial Infarction, Coronary Artery Revascularization, or Acute Stroke, Evaluating Mothers Whose Infants Had a Multi-Organ or Single-Organ Major Congenital Anomaly and Those in the Comparison Cohort (Additional Analysis 2)

eReferences

This supplementary material has been provided by the authors to give readers additional information about their work.

eTable 1. Diagnostic and procedural codes used in the study, with relevant references for validation studies.

EXPOSURE

1. Major Congenital Anomalies[1,2] by Organ System (based on 2014 EUROCAT categorization of subgroups of congenital anomalies)

	ICD8	ICD10
All congenital anomalies	740-759, *excluding* the following minor congenital anomalies<br><br>Exclusions: 745.19-745.99, 747.09 (if gestational age < 37 weeks and no other congenital heart disease), 750.09, 750.19, 751.09, 752.10 - 752.19, 752.84, 755.69, 756.81, 756.29, 756.30, 756.31, 765.39, 757.10, 757.11, 757.12, 757.13, 757.18<br><br>Also include: 761.39 (congenital rubella) and 761.49 (congenital toxoplasmosis)	All Q chapter *excluding* the following minor congenital anomalies<br><br>Exclusions: Q671, Q674, Q672, Q189, Q670, Q673, Q753, Q135, Q101, Q102, Q752, Q103, Q105, Q170, Q171, Q172, Q173, Q174, Q175, Q179, Q180, Q181, Q182, Q189, Q385, Q186, Q382, Q184, Q187, Q185, Q674, Q381, Q189, Q680, Q740G, Q845, Q8280, Q653-656, Q668, Q669, Q665, Q845, Q663, Q666, Q662, Q667, Q664, Q833, Q825, Q766, Q765, Q683, Q684, Q685, Q675, Q764L, Q676, Q682A, Q677, Q678, Q760, Q767C, Q270, Q250 (if GA < 37 weeks), Q211C, Q256 (if GA < 37 weeks), Q261, Q254E, Q331, Q314, Q315, Q320, Q401, Q430, Q400, Q633, Q610, Q627, Q552F, Q527, Q525, Q523, Q552, Q53, Q899, Q950, Q951<br><br>Q53 and Q65 specifically excluded due to poor validity in Danish National Patient Register<br>Also include (add) D215 (benign neoplasm of pelvis), D821 (DiGeorge Syndrome), D1810 (lymphangioma), P350, P351, P371

© 2018 Cohen E et al. *JAMA Network Open.*

Major Congenital Anomalies[1,2] by Organ System - *Continued*		
	**ICD8**	**ICD10**
NERVOUS SYSTEM	740-743.99	Q00-Q07
EYE	744-744.99	Q10-Q15 (exclude Q101-Q103, Q105, Q135)
EAR, FACE and NECK	745.0-745.09, (exclude 745.19-745.99, 756.81)	Q16-Q18 (exclude Q170-Q175, Q179, Q180-Q182, Q184-Q187, Q189)
CONGENITAL HEART DISEASE	746, 747 (for 747.09 (patent ductus arteriosus), exclude if gestational age < 37 weeks and no other congenital heart disease)	Q20-Q26 (exclude Q250 (if GA < 37 weeks), Q211C, Q256 (if GA < 37 weeks), Q254E, Q261
RESPIRATORY	748	Q30-34 (exclude Q314, Q315, Q320, Q331)
ORO-FACIAL CLEFT	749	Q35-Q37
DIGESTIVE SYSTEM	750.2-750.99 [exclude 750.09 (tongue anomalies) and 750.19 (pyloric stenosis)], 756.80	Q38-Q45, Q790 (exclude Q381, Q382, Q385, Q400, Q401, Q430)
ABDOMINAL WALL DEFECTS	751.1 – 751.99 [exclude 751.09 (Meckel's diverticulum)]	Q792, Q793, Q795
URINARY	752 (exclude 752.10-752.19)	Q60-Q64, Q794 (exclude Q610, Q627, Q633)
OTHER EXCLUSIONS		Q760, Q764L, Q765, Q766, Q767C, Q825, Q8280, Q833, Q899, Q950, Q951, Q845
OTHER ANOMALIES/ SYNDROMES	As below	As below
Skeletal Dysplasia	756.48, 756.49, 756.59, 756.60, 756.4	Q740B, Q77, Q780, Q782-Q788
Craniosynostosis	756.00	Q750
Congenital constriction bands/amniotic bands	No code	No code
Situs Inversus	759.00-759.09	Q893
Conjoined Twins	759.19	Q894
Congenital skin disorders	757.20-757.99	Q80-Q82
VATER/VACTERL	No code	Q872G

Major Congenital Anomalies[1,2] by Organ System - *Continued*		
	**ICD8**	**ICD10**
Vascular Disruption Anomalies	751.11, 751.19, 755.24-755.25, 755.49, 755.28, 755.31, 751.82, 756.89	Q411, Q412, Q418, Q710, Q712, Q713, Q720, Q722, Q723, Q730, Q793, Q795, Q798S, Q870G
Laterality Anomalies	745.89, 748.69, 759.00, 759.01, 759.09	Q206, Q240, Q890, Q893
Teratogenic syndromes with malformations	761.39	Q86, P350, P351, P371
Fetal Alcohol Syndrome	No code	Q860
Valproate syndrome	No code	No code
Maternal infections resulting in malformations	761.39	P350, P351, P371
Genetic Syndromes and Microdeletions	753.10	Q447B, Q619A, Q751, Q754, Q87, Q936, D821
Chromosomal	759	Q90-Q92; Q93, Q96-99

Note: At least 1 code in two or more organ systems required for inclusion as multi-organ major congenital anomalies.

The ascertainment of congenital anomalies in Danish hospital registries has predictive value (in %) of 88.2 (95% CI: 85.9, 90.5)] and completeness (in %) of 89.9 (95% CI: 87.7, 92.1).[1]

OUTCOMES

	ICD8	ICD10	Procedure/Surgery Codes
**Acute myocardial infarction**	410	I21, I22, I23	
**Stroke**[3]	43100, 43108, 43109,43190, 43198, 43199, 433, 434, 43601, 43609, 43690	I60, I61, I63 (excluding I63.6), I64, H34.1	
**Percutaneous coronary intervention**[4]			30350, 30354, 30240
**Coronary Artery Bypass Graft**[4]			30009, 30019, 30029, 30039, 30049, 30059, 30069, 30079, 30089, 30099, 30109, 30119, 30120, 30129, 30139, 30149, 30159, 30169, 30179, 30189, 30199, 30200
**Unstable angina**	No Code[3]	I20	
**Congestive heart failure**	427.09-427.11, 427.19, 428.99, 782.49	I50	
**Atrial fibrillation**[5]	427.93, 427.94	I48	
**Peripheral artery disease**[6]	443.89-443.99	I70.2, I73.9, I74.3, I74.4	
**Ischemic heart disease (non-MI)**	411-414	I20, I24, I25	
**Abdominal aortic aneurysm**	441	I71.3, I71.4	

For the primary cardiovascular disease composite outcome, the positive predictive value of each outcome using data in the Danish National Patient Registry is: myocardial infarction 97% (95% CI: 91, 99),[7] coronary artery bypass graft surgery 98% (95% CI: 93, 99],[4] percutaneous coronary intervention 98% (95% CI: 93, 99),[4] and stroke 69.3% (95% CI 67.8, 70.9).[8]

COVARIATES

1. Diabetes Mellitus[9]

Type	ICD 8	ICD 10
1 and 2	249, 250	O240, O241
Gestational	N/A	O244, O249
Unspecified	N/A	E12, E13, E14, O242, O243

2. Modified Charlson Comorbidity Index[10]

Conditions and Weight		ICD 8	ICD 10
Myocardial infarction	Weight 1	410	I21;I22;I23
Congestive heart failure	Weight 1	427.09; 427.10; 427.11; 427.19; 428.99; 782.49	I50; I11.0; I13.0; I13.2
Peripheral vascular disease	Weight 1	440; 441; 442; 443; 444; 445	I70; I71; I72; I73; I74; I77
Cerebrovascular disease	Weight 1	430-438	I60-I69; G45; G46
Dementia	Weight 1	290.09-290.19; 293.09	F00-F03; F05.1; G30
Chronic pulmonary disease	Weight 1	490-493; 515-518	J40-J47; J60-J67; J68.4; J70.1; J70.3; J84.1; J92.0; J96.1; J98.2; J98.3
Connective tissue disease	Weight 1	712; 716; 734; 446; 135.99	M05; M06; M08; M09; M30; M31; M32; M33; M34; M35; M36; D86
Ulcer disease	Weight 1	530.91; 530.98; 531-534	K22.1; K25-K28
Mild liver disease	Weight 1	571; 573.01; 573.04	B18; K70.9; K71; K73; K74; K76.0
Diabetes without end-organ damage	Weight 1	249.00; 249.06; 249.07; 249.09; 250.00; 250.06; 250.07; 250.09	E10.0, E10.1; E10.9; E11.0; E11.1; E11.9
Diabetes with end-organ damage	Weight 2	249.01-249.05; 249.08; 250.01-250.05; 250.08	E10.2-E10.8, E11.2-E11.8
Hemiplegia	Weight 2	344	G81; G82
Moderate to severe renal disease	Weight 2	403; 404; 580-583; 584; 590.09; 593.19; 753.10-753.19; 792	I12; I13; N00-N05; N07; N11; N14; N17-N19; Q61
Non-metastatic solid tumour	Weight 2	140-194	C00-C75
Leukaemia	Weight 2	204-207	C91-C95
Lymphoma	Weight 2	200-203; 275.59	C81-C85; C88; C90; C96
Moderate to severe liver disease	Weight 3	070.00; 070.02; 070.04; 070.06; 070.08; 573.00; 456.00-456.09	B15.0; B16.0; B16.2; B19.0; K72; K76.6; I85
Metastatic cancer	Weight 6	195-198; 199	C76-C80
AIDS	Weight 2	079.83	B21-B24

Note: ICD-10 codes K70.0-K70.3 (mild liver disesase) and K70.4 (moderate to severe liver disease) and ICD-8 codes removed as these are liver diseases associated with alcohol-related disesase.

Charlson Comorbidity Index codes removed for all analyses as the analysis utilized separate covariates for diabetes, alcohol-related liver disease and chronic hypertension:

a) Diabetes without end-organ damage (ICD8: 249.00; 249.06; 249.07; 249.09; 250.00; 250.06; 250.07; 250.09; ICD10: E10.0, E10.1; E10.9; E11.0; E11.1; E11.9)
b) Diabetes with end-organ damage (ICD8: 249.01-249.05; 249.08; 250.01-250.05; 250.08; ICD10: E10.2-E10.8, E11.2-E11.8)
c) Liver disease associated with alcohol use [K70.0-K70.3 (mild liver disesase) and K70.4 (moderate to severe liver disease)].
d) Hypertension-related (403, 404 in ICD8 and I11, I12, I13 and I674 in ICD10)

Codes within the Charlson Comorbidity Index that are part of specific outcome measures are excluded as well. For instance, myocardial infarction (MI) is included in CCI when analyzing stroke as an outcome, but excluded from CCI in all the analyses which include MI as an outcome (because all patients with prior MI are excluded in MI analyses).

3. Hypertension[11]

ICD 8	ICD 10
400-404	I10-I15, I674

4. Alcohol-Related Disease[3]

ICD 8	ICD 10
291, 303, 577.10, 979, 980	G312, G621, G721, I426, K292, K860, R780, Z721, F10, T51, K70

5. Spontaneous Abortion[12]

	ICD 8	ICD 10
Missed Abortion	6346x	O021, O021A
Missed Abortion with dilation and curettage	6451x	
Spontaneous Abortion		O03x
Spontaneous Abortion, other	6438x	
Spontaenous Abortion with complications	6439x	

6. Pregnancy Complications[11,13-17]

	ICD 8	ICD 10
<u>A) Placental</u>		
Pre-eclampsia	63703, 63704, 63709	O140—O142, O149, O150-O159
Gestational or unspecified Hypertension	63700	O13, O16
Placental abruption	63219, 6515	O45
Placental infarction	No code	O43.1, O43.8, O43.9
<u>B) Non-Placental</u>		
Intrauterine hypoxia and birth asphyxia	77640–77650, 76840, 76890	P20, P21
Uterine rupture	659	O71.0, O71.1
Umbilical cord prolapse or vasa previa	663.3, 663.4	O69

Amniotic fluid embolism	673.1	O88.1
Fetal-maternal hemorrhage	No code	O43.0
Chorioamnionitis	No code	O41.1

Other Notes on Covariates:

Marital status was ascertained from Danish Civil Registry at the time of the index event. A 6 month allowance was made post-index event to allow for delayed registration of marriage.

eTable 2. Development of the cardiovascular disease composite outcome of hospitalization for acute myocardial infarction, coronary artery revascularization, or acute stroke, evaluating mothers whose infant had a major congenital anomaly with matching dissolved stratified by duration of follow-up *(Additional analysis 6)*.

	**No. of events/women at risk**		**Incidence rate per 1000 person-years (95% CI)**		**Hazard ratio (95% CI)**		
	**Exposed**	**Comparison**	**Exposed**	**Comparison**	**Rate difference**	**Unadjusted**	**Adjusted[a]**
Additional analysis 6:							
**Year of Birth**							
1979-1993	736/20,232	6,218/202,133	1.37 (1.27-1.47)	1.15 (1.12-1.18)	0.22 (0.11-0.32)	1.20 (1.11-1.29)	1.14 (1.05-1.23)
1994-2003[b]	142/11,178	1,083/111,605	0.86 (0.72-1.01)	0.66 (0.62-0.70)	0.21 (0.06-0.35)	1.31 (1.10-1.57)	1.13 (0.95-1.35)
2004-2013[c]	36/11,483	215/114,313	0.64 (0.43-0.85)	0.38 (0.33-0.43)	0.26 (0.04-0.47)	1.67 (1.17-2.38)	1.44 (1.01-2.06)

[a] Adjusted for matching factors used in primary analysis (parity, mother´s age, and year of child´s birth), maternal demographics (marital status, immigration status), socio-economic status (income quartile, educational level), previous maternal health (diabetes mellitus, modified Charlson Comorbidity Index score, chronic hypertension, history of alcohol-related disease, depression), previous spontaneous abortion, and pregnancy complications.

[b] Additional covariate adjusted for in 1994-2003 includes maternal smoking history. Without this additional covariate, the adjusted hazard ratio (95% CI) is 1.15 (0.97-1.38).

[c] Additional covariates adjusted for in 2004-2013 include maternal smoking history and body mass index. Without these additional covariates, the adjusted hazard ratio (95% CI) is 1.47 (1.03-2.10).

eFigure. Development of the cardiovascular disease composite outcome of a hospitalization for acute myocardial infarction, coronary artery revascularization, or acute stroke, evaluating mothers whose infants had a multi-organ or single-organ major congenital anomaly and those in the comparison cohort (*Additional analysis 2).*

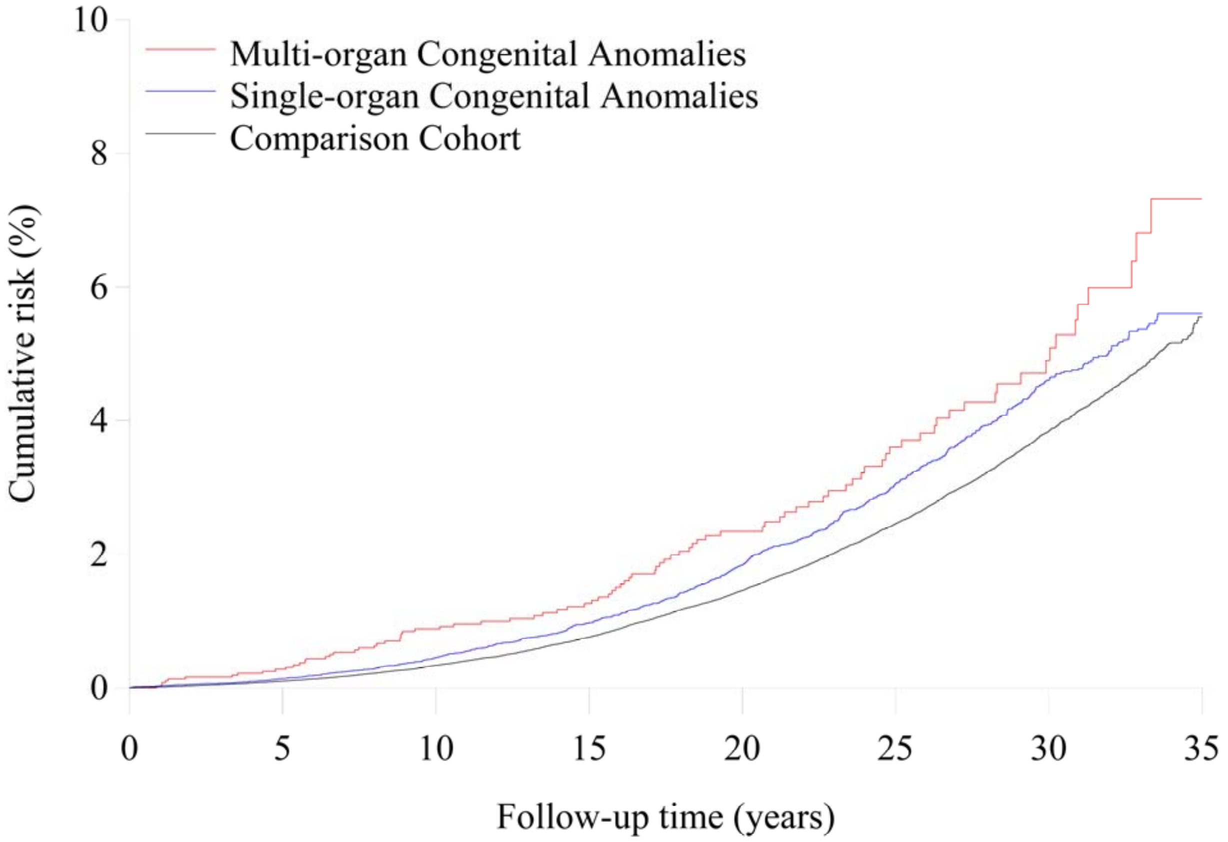

Number at risk:

Multi-organ	3837	3254	2652	2043	1435	941	477
Single-organ	39056	33420	28076	22959	17783	11533	5611
Comparison	428051	366143	307667	251237	193625	125817	61778

eReferences

1. Larsen H, Nielsen GL, Bendsen J, Flint C, Olsen J, Sorensen HT. Predictive value and completeness of the registration of congenital abnormalities in three Danish population-based registries. *Scand J Public Health.* 2003;31(1):12-16.
2. Pasternak B, Svanstrom H, Molgaard-Nielsen D, Melbye M, Hviid A. Metoclopramide in pregnancy and risk of major congenital malformations and fetal death. *JAMA.* 2013;310(15):1601-1611.
3. Pottegard A, Garcia Rodriguez LA, Poulsen FR, Hallas J, Gaist D. Antithrombotic drugs and subarachnoid haemorrhage risk. A nationwide case-control study in Denmark. *Thromb Haemost.* 2015;114(5):1064-1075.
4. Adelborg K, Sundboll J, Munch T, et al. Positive predictive value of cardiac examination, procedure and surgery codes in the Danish National Patient Registry: a population-based validation study. *BMJ Open.* 2016;6(12):e012817.
5. Frost L, Vestergaard P. Caffeine and risk of atrial fibrillation or flutter: the Danish Diet, Cancer, and Health Study. *Am J Clin Nutr.* 2005;81(3):578-582.
6. Sogaard KK, Schmidt M, Pedersen L, Horvath-Puho E, Sorensen HT. 30-year mortality after venous thromboembolism: a population-based cohort study. *Circulation.* 2014;130(10):829-836.
7. Sundboll J, Adelborg K, Munch T, et al. Positive predictive value of cardiovascular diagnoses in the Danish National Patient Registry: a validation study. *BMJ Open.* 2016;6(11):e012832.
8. Luhdorf P, Overvad K, Schmidt EB, Johnsen SP, Bach FW. Predictive value of stroke discharge diagnoses in the Danish National Patient Register. *Scand J Public Health.* 2017;45(6):630-636.
9. Wu CS, Nohr EA, Bech BH, Vestergaard M, Olsen J. Long-term health outcomes in children born to mothers with diabetes: a population-based cohort study. *PLoS One.* 2012;7(5):e36727.
10. Thygesen SK, Christiansen CF, Christensen S, Lash TL, Sorensen HT. The predictive value of ICD-10 diagnostic coding used to assess Charlson comorbidity index conditions in the population-based Danish National Registry of Patients. *BMC Med Res Methodol.* 2011;11:83.
11. Hansen AT, Schmidt M, Horvath-Puho E, et al. Preconception venous thromboembolism and placenta-mediated pregnancy complications. *J Thromb Haemost.* 2015;13(9):1635-1641.
12. Lohse SR, Farkas DK, Lohse N, et al. Validation of spontaneous abortion diagnoses in the Danish National Registry of Patients. *Clin Epidemiol.* 2010;2:247-250.
13. Ray JG, Redelmeier DA, Urquia ML, Guttmann A, McDonald SD, Vermeulen MJ. Risk of cerebral palsy among the offspring of immigrants. *PLoS One.* 2014;9(7):e102275.
14. Laszlo KD, Liu XQ, Svensson T, et al. Psychosocial stress related to the loss of a close relative the year before or during pregnancy and risk of preeclampsia. *Hypertension.* 2013;62(1):183-189.

15. Altman M, Edstedt Bonamy AK, Wikstrom AK, Cnattingius S. Cause-specific infant mortality in a population-based Swedish study of term and post-term births: the contribution of gestational age and birth weight. *BMJ Open.* 2012;2(4).
16. Auger N, Fraser WD, Healy-Profitos J, Arbour L. Association Between Preeclampsia and Congenital Heart Defects. *JAMA.* 2015;314(15):1588-1598.
17. Rai R, Regan L. Recurrent miscarriage. *Lancet.* 2006;368(9535):601-611.
